# Mentalizing impairments in somatic symptom disorder: a systematic review and meta-analysis

**DOI:** 10.3389/fpsyg.2026.1704193

**Published:** 2026-02-13

**Authors:** Krisztina Kocsis-Bogar, Judit Deri, Mónika Miklósi, Brigitte Wildner, Alexander Kaltenboeck, Marion C. Aichberger

**Affiliations:** 1 Clinical Division of Social Psychiatry, Department of Psychiatry and Psychotherapy, Medical University of Vienna, Vienna, Austria; 2 Comprehensive Center for Clinical Neurosciences and Mental Health, Medical University of Vienna, Vienna, Austria; 3 Department of Developmental and Clinical Child Psychology, Faculty of Education and Psychology, Eötvös Loránd University, Budapest, Hungary; 4 University Library, Medical University of Vienna, Vienna, Austria

**Keywords:** mentalizing, social cognition, somatic symptom disorder, somatization, somatoform, theory of mind, pain

## Abstract

**Background:**

Mentalizing deficits have been observed across various psychiatric disorders, but there is currently no systematic review or meta-analysis about the differences of mentalizing performance in patients with somatic symptom disorder (SSD) compared to non-clinical or clinical controls. The aim of the present paper is to fill this gap.

**Method:**

A systematic search of databases, including Embase, MEDLINE, CENTRAL, PsycInfo, Psyndex, LILACS, Scopus, Web of Science, OpenGrey and Open Doar was conducted up to June 2025.

**Results:**

Altogether 3,796 records were identified and screened according to the PRISMA guidelines. Ten cross-sectional case-controls studies in patients with SSD and controls (12 publications, *N* = 314) were found eligible for our review, out of which nine studies were included in our meta-analysis. High risk of bias and modest methodological quality was found in the majority of the studies using the AHRQ method. SSD patients demonstrated significant impairments in mentalizing in general as well as in mentalizing about cognitive and affective states (*p* = 0.001) compared to non-clinical controls. A homogeneous effect of undermentalizing (*p* = 0.007) was observed compared to non-clinical controls, though no significant mentalizing differences were found between the observed patient group and patients with major depressive disorder (MDD) or posttraumatic stress disorder (PTSD).

**Conclusion:**

Patients with SSD exhibit reduced mentalizing performance compared to non-clinical controls, but no mentalizing deficit could be shown compared to those with MDD or PTSD, likely due to the limited number and methodological heterogeneity of relevant studies.

**Systematic review registration:**

https://www.crd.york.ac.uk/prospero/display_record.php?RecordID=462714

## Introduction

Social cognition is a broad area encompassing abilities to perceive, interpret, and process information in the interpersonal context, by perception meaning the recognition of social roles, rules and other important cues in the social context (Green et al., 2019), as well as the attribution of thoughts and emotions to other individuals (Lane and Smith, 2021). Specifically, mentalizing is defined as the ability to infer the mental states of others and to anticipate their behavior, as well as one’s own (Premack and Woodruff, 1978). It encompasses amongst others the attribution of knowledge, intentions, perceptions and emotions to the self and others (Quesque et al., 2024).

Mentalizing plays an essential role in human social interactions (Frith and Frith, 1999). Mentalizing impairments can lead to difficulties in everyday social settings, which potentially manifest themselves in form of insufficient regulation of aggression and behavioural abnormalities. These can impact existing social relationships and make it more challenging to build up new ones (Henry et al., 2016). In previous studies—mainly concentrating on schizophrenia—two main types of mentalizing errors were described: under- and overmentalizing. In case of undermentalizing errors, individuals are suggested to lack a comprehensive theory of mind, consequently, they make overly simplistic mental state inferences (Frith, 2004; Montag et al., 2011). Those, with overmentalizing, however, use a false and overly complex mental state reasoning (Frith, 2004; Montag et al., 2011).

Social cognition is considered to be an advanced neurocognitive process depending on the executive functions (Court et al., 2025) and is known to rely on overlapping brain areas with information-processing, inhibition, organizing and planning future actions, anticipation, decision-making and working memory (Adolphs, 2001; Green et al., 2015; Tanji and Hoshi, 2001). The awareness of one’s own emotions is regarded as a further prerequisite for the successful recognition of the emotional expressions of others and consequently, of accurate mentalizing (Lane and Smith, 2021), whereas the lack of this awareness—also defined as alexithymia—means the difficulty or inability to recognize, distinguish and verbally describe one’s own emotions (Sifneos, 1991).

There is an abundance of tools used to measure mentalizing ability in adults. Reading the Mind in the Eye Task [RMET] (Baron-Cohen et al., 2001), Movie for the Assessment of Social Cognition [MASC] (Dziobek et al., 2006), and Animation Task [AT] (Abell et al., 2000) are among the most commonly used mentalizing tasks for adults, with at least acceptable internal consistency, good known-group, convergent and discriminant validity, RMET also with good criterion validity. AT has demonstrated acceptable inter-rater reliability, although test–retest reliability data are not available (Yeung et al., 2024). Levels of Emotional Awareness Scale [LEAS], (Lane et al., 1990) is primarily regarded a measure of emotional awareness, however, in the “Other” condition participants are required to describe how someone else would feel in a series of complex emotional situations, which is a key aspect of mentalizing about affective states. Therefore, in line with Lane et al. (2015), LEAS “Self” is seen as a task to measure emotional awareness, LEAS “Other” subtest is considered to be a mentalizing task. Further, a common self-report measure of mentalizing ability should be mentioned: the Perspective Taking subscale of the Interpersonal Reactivity Index (Davis, 1983), however the predictive validity of self-report measures regarding mentalizing performance is questionable (Murphy and Lilienfeld, 2019). For a comprehensive review of a wider range of mentalizing tasks, please see the work of Yeung et al. (2024).

The mentioned tools are very different in relying on emotion recognition in the process of mentalizing. For example, RMET (Baron-Cohen et al., 2001), has been criticized for relying too heavily on emotion recognition rather than directly measuring mentalizing ability (Oakley et al., 2016), which is especially relevant for individuals with high alexithymia, potentially including those with somatic symptom and related disorders (Di Tella et al., 2020). In contrast, other tools do not involve emotion recognition at all. In the case of AT (Abell et al., 2000), for example, participants observe geometrical figures’ movements and in case of LEAS (Lane et al., 1990) participants are required to describe how they and a potential other person would feel in an imaginary situation. MASC, on the other hand, is a video-based tool showing the whole complexity of a series of social situations, including gestures and the tone of voice additionally to facial expressions. A further advantage of MASC is that it measures the types of mentalizig mistakes too, such as under- and overmentalizing (Dziobek et al., 2006).

Impairments of mentalizing ability have been demonstrated across a variety of psychiatric conditions, such as autism (Ozbek et al., 2023) schizophrenia (Eriksdotter et al., 2023), social anxiety disorder (Alvi et al., 2022), depression (Pagnoni et al., 2022), bipolar disorder (Bora et al., 2015) eating disorders (Caglar-Nazali et al., 2014), body dysmorphic disorder (Court et al., 2025), and borderline personality disorder (Németh et al., 2018), in comparison to non-clinical controls. In psychiatric disorders, mentalizing impairment has been linked with symptom severity (Johnson et al., 2022). Although there is evidence of impaired mentalizing ability in patients with different types of somatoform (Thamby et al., 2019), somatization (Zunhammer et al., 2015), or somatic symptom disorder (SSD) (Cetin et al., 2021), no systematic reviews or meta-analyses on this topic are available, to our knowledge. Our aim is to fill this gap by critically assessing the current literature concerning mentalizing performance in these patient groups compared to non-clinical and clinical controls.

There are several reasons for expecting impaired mentalizing in SSD compared to non-clinical controls, one of these being the impairment of emotional awareness (EA) in SSD (Yan et al., 2024), which would suggest difficulties in mentalizing about the emotional states of others. However, it is unclear if diminished EA is connected with impaired mentalizing. Some studies (Lane et al., 2015; Subic-Wrana et al., 2010a), particularly those using RMET (Baron-Cohen et al., 2001) have found this link, whereas others, using MASC (Dziobek et al., 2006) and relying less on emotion recognition, have failed to do so (Pisani et al., 2021). Additionally, the impairment of EA may not be specific for SSD. Traditionally, alexithymia was connected with somatoform or somatization disorders, but recently it has been recognized as a transdiagnostic phenomenon (von Schoenhueb et al., 2023) and has been identified in multiple psychiatric disorders (Morie et al., 2022).

Apart from EA, some further factors with relevance for the mentalizing ability in SSD must be mentioned, such as shortcomings of executive functions and the reduction of social motivation and engagement (Apperly, 2012). There is evidence of attention and executive function impairments in individuals with undifferentiated somatoform disorders (Al-Adawi et al., 2010; Chutko et al., 2019), SSD (Chutko et al., 2019; de Vroege et al., 2018; Wu et al., 2023) or body dysmorphic disorders (Court et al., 2025; Rajabi et al., 2022), which are understandable based on the cognitive overload of health-related or body-related concerns (Kube et al., 2023).

As regards to social motivation and engagement, individual differences in mentalizing ability and social motivation have been found to correlate from early adolescence on (Devine and Apperly, 2022). Individuals with lower mentalizing performance may struggle in social situations requiring cooperation (Markiewicz et al., 2023). Similarly, SSD patients have been described as having cooperation difficulties in healthcare settings and are often seen as “difficult” by healthcare professionals (Brown, 2007). They are often presenting with a combination of potentially interrelated issues, such as psychiatric comorbidities, attachment problems, personal vulnerability and externalizing behaviors (Hijne et al., 2022), tend to overuse healthcare services (Barsky et al., 2005), yet feel frustrated and dissatisfied with the care they receive (Henningsen, 2018). Difficulties of social interactions in health-care and private settings may contribute to social isolation, negative self-perception depressive and anxious symptoms as well as a lower level of general functioning and lower quality of life in SSD (Löwe et al., 2022), some of which have been shown to contribute to the persistence of SSD (Limburg et al., 2017).

Although it seems to be logical to expect impairments in mentalizing in patients with SSD compared to non-clinical controls based on the previous arguments, it is unclear, if specific mentalizing problems distinctive of SSD can be found compared to any other psychiatric conditions. In line with the transdiagnostic view of mentalizing impairments (Nestor et al., 2024; Pisani et al., 2021), mentalizing deficits could be present to a similar extent across the whole range of different psychiatric disorders. Further, there is a high comorbidity of SSD and other psychiatric disorders, such as borderline personality disorder (Links et al., 2025), depression, bipolar and anxiety disorders, post-traumatic stress disorder, substance use and different types of eating disorders (Gureje et al., 2025; Ionescu et al., 2021; Wu et al., 2023), which can complicate the comparison of SSD and any of these psychiatric conditions. It would require a systematic assessment of comorbidities to make this comparison and to examine the effect of them on mentalizing impairments.

Depression has been reported to be the most studied comorbidity in SSD (Wu et al., 2023), therefore it will be part of the focus of our review. Both of the previously mentioned functional difficulties, such as the executive function deficits (Pagnoni et al., 2022) as well as the diminished social interest and engagement (Kupferberg and Hasler, 2023) are also known in patients with depression. Given these findings, it would be reasonable to expect impaired mentalizing ability in patients with SSD due to their depressive vulnerability too.

The current review aims to explore the following two research questions:

1. Do patients with SSD and corresponding diagnoses show impaired mentalizing in comparison to non-clinical controls and patients with any other psychiatric disorder?
2. Is this impairment independent of age, sex or comorbid depressive symptoms?

## Materials and methods

The systematic review was performed according to the Preferred Reporting Items for Systematic Reviews and Meta-Analyses (PRISMA) guidelines (Liberati et al., 2009) and registered on PROSPERO (CRD42023462714) prior to data analysis.

### Selection criteria and search strategy

#### Participant characteristics

Studies involving patients aged 18 years and above with SDD according to DSM-5 (APA, 2013), bodily distress disorder according to ICD-11 (WHO, 2019). Corresponding diagnoses from the previous manuals, such as somatoform, undifferentiated somatoform and persistent somatoform pain disorder according to ICD-10 (WHO, 1993) as well as somatization, undifferentiated somatization and pain disorder according to DSM-IV-TR (APA, 2000) were included. The eligible diagnoses are presented in detail in Table 1. No restrictions were applied regarding sex or culture.

**Table 1:** Eligible ICD-10, DSM-IV, and DSM-5 diagnoses for the present systematic review and meta-analysis.

Somatization and related disorders in the different diagnostic classification systems				Eligible for the current review?	
ICD-10	ICD-11	DSM-IV	DSM-5	YES	NO
Somatoform disorder	Bodily distress disorder	Somatization disorder	Somatic symptom disorder	X	
Undifferentiated somatoform disorder		Undifferentiated somatization disorder		X	
Persistent somatoform pain disorder		Pain disorder		X	
Hypochondriacal disorder		Hypochondriasis	Illness anxiety disorder		X
Somatoform autonomic disorder other somatoform disorders			Other specified somatic symptom disorder and related disorders		X
Somatoform disorder unspecified		Somatoform disorder not otherwise specified	Unspecified somatic symptom and related disorders		X
Body dysmorphic disorder		Body dysmorphic disorder			X
Dissociative and conversion disorders		Conversion disorder	Conversion disorder		X

Studies using non-clinical and clinical control groups were included regardless of diagnostic method, symptom severity or treatment setting. We restricted our review to in- and outpatients with established diagnoses, excluding studies including only non-clinical individuals with somatization symptoms, as we expected to find clearer differences compared to controls in this population. Studies of patients with psychiatric comorbidities, other than psychotic symptoms in the last month, were included.

#### Intervention

Studies were included if they used measures of mentalizing, which were consistently administered, scored, and interpreted to allow meaningful comparisons across populations or time points.

#### Comparison

Studies were deemed eligible only if they also included a non-clinical control group or one with another psychiatric or a somatic condition.

#### Outcome

Studies measuring differences in mentalizing about cognitive or affective states or both in patients relative to the control groups were included.

The literature search was conducted in June 2025, encompassing studies in any language, publication time or publication status. The search covered Embase, MEDLINE via Ovid, Cochrane Central Register of Controlled Trials (CENTRAL) via Ovid, PsycInfo via Ovid, Psyndex via Ovid, LILACS, Scopus, Web of Science Core Collection. Additional search of grey literature was conducted using OpenGrey and Open Doar. The databases were systematically explored using search terms tailored for each database. The detailed search strategy (constructed by an Academic Research Librarian) is provided in Supplementary material 1. It included controlled vocabulary (MeSH terms) and free keywords. Reference lists of the relevant papers were searched through manually and an additional forward search of the literature citing the relevant papers was conducted but yielded no additional results.

### Study selection

**Figure 1:**
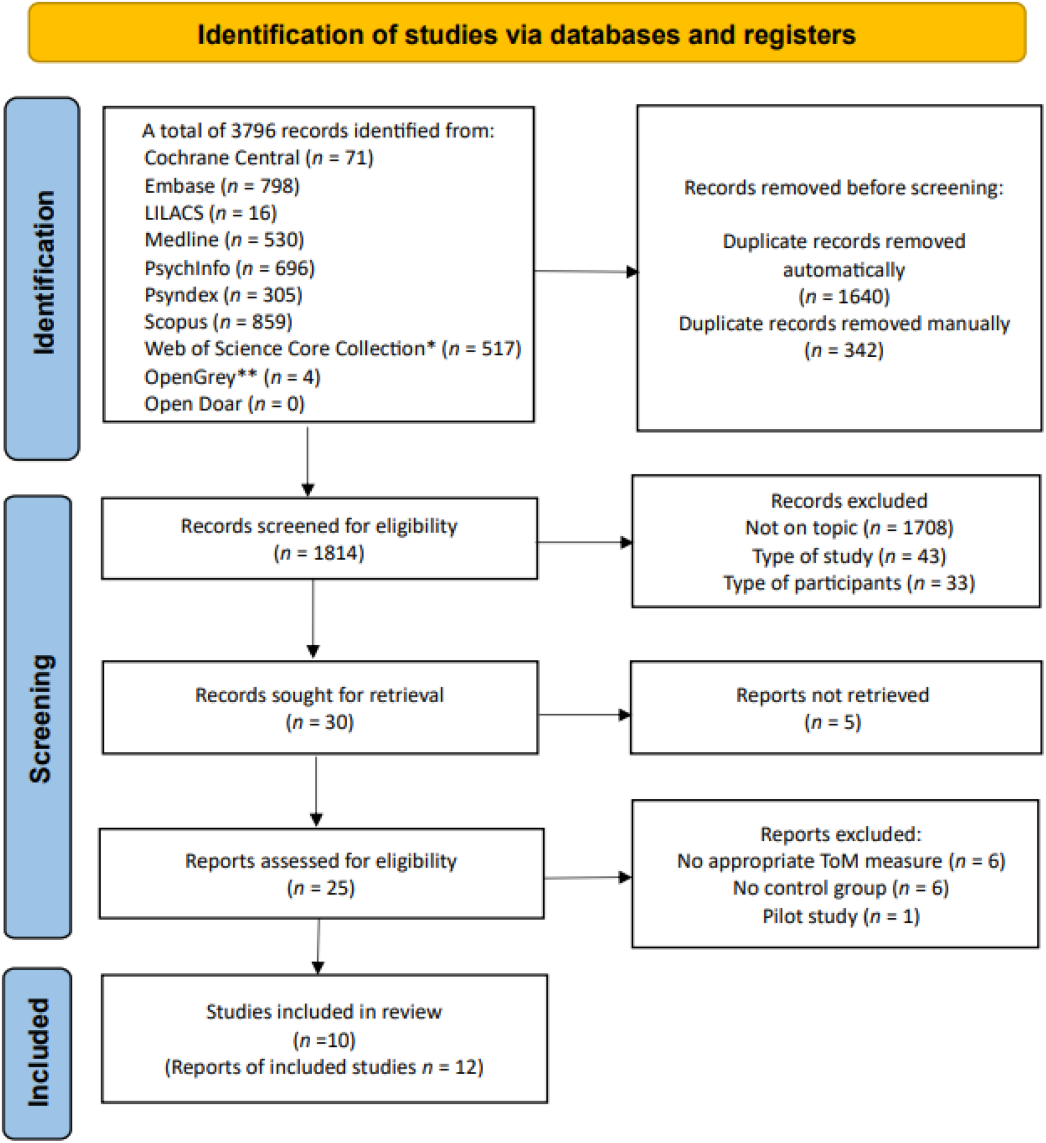
Study selection process according to the PRISMA guidelines. *Including the following collections: Social Sciences Citation Index (SSCI), Science Citation Index Expanded (SCI-EXPAND), Emerging Sources Citation Index (ESCI), Conference Proceedings Citation Index – Social Science & Humanities (CPCI-SSH), Conference Proceedings Citation Index – Science (CPCI-S), Book citation Index –Science (BKCI-S), Arts & Humanities Citation Index (A&HCI), Book Citation Index – Social Sciences & Humanities (BKCI-SSH). **DANS Data Station Social Sciences and Humanities and Life Sciences were searched.

The screening process is summarized in the flowchart (Figure 1) following the PRISMA statement (Page et al., 2021). After removing duplicates (both automatically and manually the two primary review authors (KKB and JD) screened the remaining titles and abstracts for eligibility (Higgins et al., 2023). Only case–control studies were included. Book chapters, protocols and letters to the editor were excluded. There was a substantial agreement between the two review authors about the potentially eligible titles and abstracts (*K* = 0.75, *p* < 0.001). In the next phase, the relevant full-text articles were retrieved and assessed for the eligibility criteria. There was an almost perfect agreement (*K* = 0.96, *p* < 0.001) between the two review authors at this phase. Discrepancies regarding the inclusion of abstracts or full texts were resolved through discussion between the two aforementioned review authors, with consultation from the supervisor (MA) if necessary.

### Data extraction

Two review authors (KKB and JD) performed data extraction independently, using an Excel extraction sheet, including the following categories: title, authors, year of publication, source, publication type, potential conflicts of interest, study design, diagnosis of patients and controls (if applicable), diagnostic methods, comorbidity, type and number of controls, dropouts, measure of mentalizing, outcomes of the comparison between patients and controls, any other results reported about the connection between somatization symptoms and mentalizing ability, key conclusions of study authors. Differences were resolved through discussion. In cases of insufficient or unclear data, study authors were contacted to clarify missing information.

### Assessment of risk of bias (ROB)

The following 11 areas were evaluated (Rostom et al., 2004): 1. Definition of the source of information; 2. List of inclusion and exclusion criteria; 3. Time period of identifying participants; 4. Indication of recruiting process as consecutive or not; 5. Indication of whether evaluators of subjective components were blinded to other aspects of the patients’ status; 6. Assessments undertaken for quality assurance purposes; 7. Explanation of any patient exclusions from the analysis; 8. Description of the assessment and control of confounding variables; 9. Explanation of handling missing data 10. Summary of patient response rate and completeness of data collection, 11. Follow-up. Overall, ROB assessment was based on study design and conduct rather than reporting, as suggested in the Agency for Healthcare Research and Quality (AHRQ) draft report (AHRQ, 2012). Study quality and bias were assessed by two authors (KKB and JD) using the AHRQ checklist (Rostom et al., 2004). Discrepancies were resolved by consensus between the two researchers. ROB management for studies fulfilling less than 4 out of 11 AHRQ criteria is considered to be “poor,” between 4 and 5 “fair,” 6 and 8 “good,” and 9 and above “excellent.”

### Statistical analyses

For the meta-analysis, the Comprehensive Meta-Analysis (Borenstein, 2009) software was used. Random effect models were applied, accounting for sampling and study-level errors. Overall effect size was calculated and reported as Hedges’ *g* value. Heterogeneity was tested using Cochrane’s *Q* and *I*^2^ statistics. Leave-one-study-out sensitivity analysis was conducted to test the stability and the influence of studies. Outliers were identified by standardized residuals above/below ± 3.29, and excluded. Publication bias was assessed using funnel plots and Rosenthal’s fail-safe *N* (Rosenthal, 1979). If bias was detected, Duval Tweedie’s methods (Borenstein, 2009) were applied for adjustment. Moderator analyses were conducted via subgroup analysis for categorical moderators (outcome, methodology, somatoform disorders diagnosis), and meta-regression analyses for publication year, study quality, mean age, the ratio of females in the sample and depressive symptoms.

## Results

### Systematic review

Our search yielded a total of 3,796 records. In the end, 10 studies (12 publications) were found eligible and included in the final review: nine journal articles and one doctoral thesis. Study characteristics are shown in Table 2. The 10 studies included 314 patients (184 women, 130 men) diagnosed with SSD or a corresponding disorder, along with 275 clinical and 271 non-clinical controls. The studies were conducted in three different countries: Germany (*n* = 8), Turkey (*n* = 1), and India (*n* = 1). Seven studies used only non-clinical controls, while three studies included controls with major depressive disorder (MDD), anxiety disorder (AD), and posttraumatic stress disorder (PTSD).

**Table 2:** Overview of the reviewed studies in chronological order.

Authors	Patients (% female)	Age (*SD*)	Diagnosic tool	Comorbidity	Control group	Country	Setting	Measure of mentalizing	Mentalizing impairment	Effect Size
Subic-Wrana et al. (2010a)	30 SFD (73.3)	39.67 (10.48)	Video-taped interviews of ICD-10 criteria	Assessed, but no scores reported	30 NC	Germany	Inpatient	LEAS, Frith-Happé AT	SFD < NC	*d* = 0.61
Subic-Wrana et al. (2010b)	42 SFD (52.4)	40.4 (11.3)	ICD-10 criteria; by physicians, psychologists	No assessment	68 MDD, 34 AD, 57 other diagnoses	Germany	Inpatient	RMET	Non-significant	–
de Greck et al. (2012)	20 SFD (60)	42.50 (14.00)	SCID-IV	Depressive symptoms	20 NC	Germany	Inpatient	IRI, Perspective taking	Non-significant	–
Noll-Hussong et al. (2013)	21 CPD (80)	46.62 (12.49)	SCID-IV	Depressive and anxiety symptoms	19 NC	Germany	Outpatient	IRI, Perspective taking	Non-significant	–
Schönenberg et al. (2014)	19 PSPD (100)	47.05 (8.92)	Semi-structured interview based on ICD-10, SCID-IV	Assessed, but no scores reported	19 NC	Germany	Inpatient	MASC	*Overall mentalizing,* PSPD < NC	η^2^ = 0.16
									*Mentalizing affective states*, PSPD < NC	η^2^ = 0.20
									*Overmentalizing* PSPD < NC	η^2^ = 0.19
Ruckmann (2015)	48 SFD (70.8)	41.81 ± 14.58	SCID-IV	Assessed, but no score reported	48 NC (50)	Germany	Outpatient	IRI, Perspective taking	Non-significant	η^2^ = 0.009
Zunhammer et al. (2015)	30 PSPD (67)	50.20 (8.60)	SCID-IV	Depressive symptoms	30 NC	Germany	Inpatient	LEAS, Frith-Happé AT	PSPD < NC	*d* = 0.69
Thamby et al. (2019)	20 SFD (50)	36.5 (9.3)	ICD-10 Criteria	Depressive and anxiety symptoms	20 NC	India	Outpatient	SOCRATIS	SFD < NC	*d* = 1.12
Cetin et al. (2021)	48 SSD (75)	39.83 (11.93)	Interview based on DSM-V	Depressive symptoms	50 MDD 50 NC	Turkey	Outpatient	RMET	SSD < MDD, NC	–
Seitz et al. (2022)	36 SSD (77.8)	30.42 (11.12)	SCID-V	Depressive symptoms, psychological distress, PTSD and trauma history	33 MDD 33 PTSD 35 NC	Germany	In- and outpatient	MASC	*Overall mentalizing*, SSD = NC, PTSD < NC	η^2^ = 0.10

SSD, somatic symptom disorder; CPD, chronic pain disorder; PSPD, persistent somatoform pain disorder; SFD, somatoform disorders; MDD, major depressive disorder; AD, anxiety disorder; PTSD, posttraumatic stress disorder; NC, non-clinical control; LEAS, Levels of Awareness Scale; AT, Animation Task; RMET, Reading the Mind in the Eye Task; MASC, Movie for the Assessment of Social Cognition; SOCRATIS, Social Cognition Rating Tool in Indian Settings.

The different measures of mentalizing used in the individual studies are summarized in Table 3.

**Table 3:** Overview of the measures used in the selected studies.

Measure	Description	Type of mentalizing	Format	Scoring
Interpersonal Reactivity Index (IRI), Perspective Taking (PT) subscale (Davis, 1983)	Self-report measure assessing tendency to adopt others’ viewpoints	Perspective taking, mentalizing about cognitive states	7-item questionnaire (incl. 2 reverse-scored items)	Total score based on a Likert-type scale
Levels of Emotional Awareness Scale (LEAS)—“Other” condition (Lane et al., 1990)	Participants describe emotional responses for self and others in 20 interpersonal scenarios	Emotional awareness and mentalizing about affective states	20 vignettes with questions	Responses scored for emotional complexity (higher = greater awareness)
Frith-Happé Animation Task (AT) (Abell et al., 2000)	Participants interpret animations of triangles in three interaction types: random, goal-directed, ToM interaction	Mentalizing about cognitive states	Visual and open-ended response	Accuracy in mental state attribution
Reading the Mind in the Eyes Task (RMET) (Baron-Cohen et al., 2001)	36 photos of eyes depicting emotions	Mentalizing about affective states	Forced-choice out of four options	Total number of correct answers
Movie for Assessment of Social Cognition (MASC) (Dziobek et al., 2006)	Video-based scenes with multiple-choice questions on mental states	Mentalizing about cognitive and affective states, Mentalizing error types	Audiovisual and multiple choice	Accuracy plus error type categorization (under- or overmentalizing, no-ToM)
Social Cognition Rating Tool in Indian Settings (SOCRATIS) (Mehta et al., 2011)	Test battery including classic mentalizing tasks (e.g., Sally-Ann, irony, faux pas) adapted for the Indian culture	Mentalizing about cognitive states	Multiple classic tasks	Task-specific scoring

Most of the included studies exhibited a relatively high ROB, though AHRQ criterion 11 (Follow-ups) was not applicable to the present cross-sectional studies (Table 4). Four studies met less than four criteria of the AHRQ assessment tool, indicating a high ROB, and, consequently, modest methodological quality. Five studies met four or five AHRQ criteria, indicating fair, and only one study met six, indicating good quality and moderate ROB.

**Table 4:** Quality rating of the selected studies according to the AHRQ criteria.

Article	Items											
	Q1	Q2	Q3	Q4	Q5	Q6	Q7	Q8	Q9	Q10	Q11	Total
Subic-Wrana et al. (2010a)	+	+	−	+	−	−	−	+	−	−	NA	4
Subic-Wrana et al. (2010b)	+	−	+	?	−	−	−	−	−	−	NA	2
De Greck et al. (2012)	+	+	−	?	−	−	−	+	−	−	NA	3
Noll-Hussong et al. (2013)	+	+	+	?	−	−	−	+	−	−	NA	4
Schönenberg et al. (2014)	+	+	−	+	−	−	+	+	−	−	NA	5
Zunhammer et al. (2015)	+	+	−	+	+	−	−	+	−	−	NA	5
Ruckmann (2015)	+	−	−	?	−	−	−	+	+	−	NA	3
Thamby et al. (2019)	+	+	−	?	−	−	−	+	−	−	NA	3
Cetin et al. (2021)	+	+	+	+	−	−	+	+	−	−	NA	7
Seitz et al. (2022)	+	+	−	+	−	−	+	+	−	−	NA	5

+, yes; −, no; ?, unclear; NA, non-applicable.

### Meta-analysis

Out of the 10 selected studies, nine were included in the meta-analysis. The study of Subic-Wrana et al. (2010b), also including controls with MDD and AD, was excluded due to the lack of exact group means for mentalizing. In total, nine studies comparing SSD patients with non-clinical controls were included in the first comparison, and two studies comparing SSD patients with those suffering from MDD or PTSD were included in the second comparison.

#### Comparison of patients with SSD or corresponding diagnoses and non-clinical controls

##### Mean effect size

**Figure 2:**
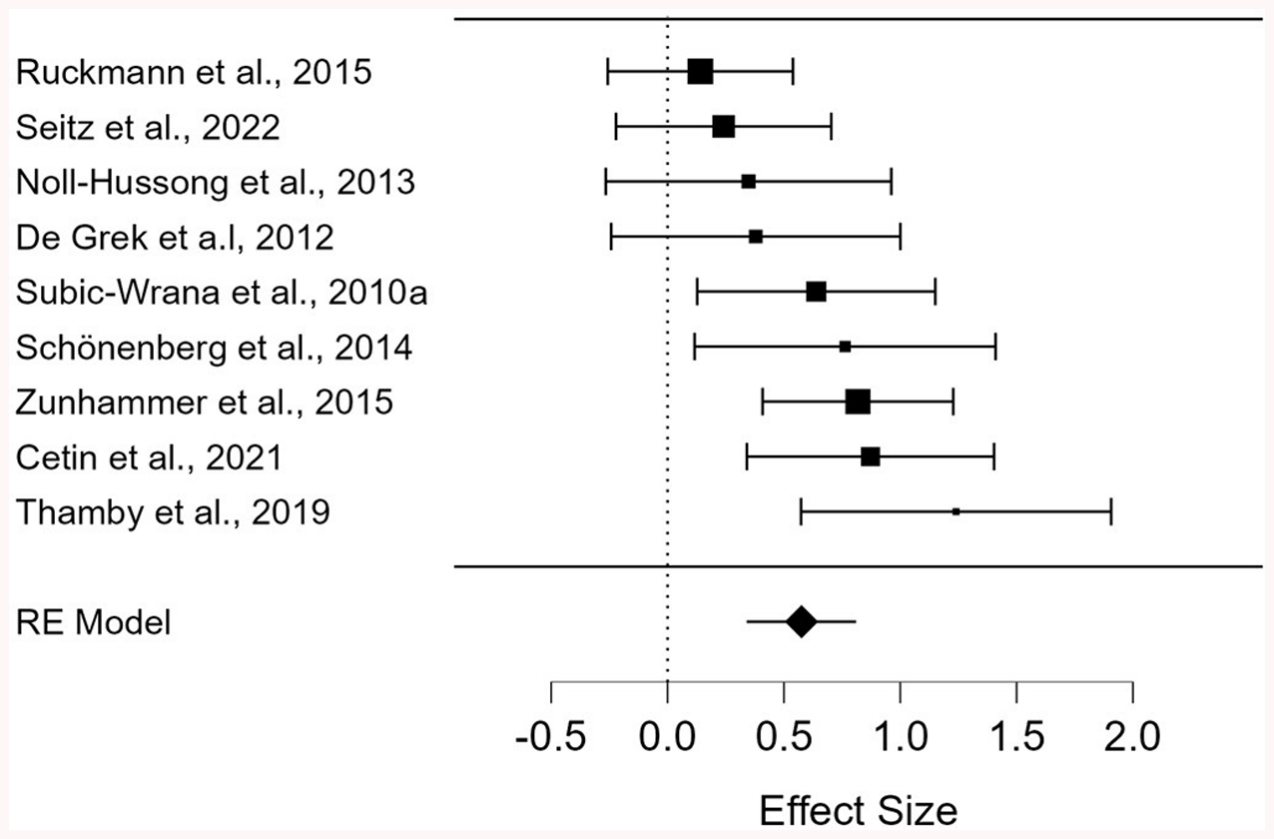
Forest plot of mentalizing differences between patients with somatization and nonclinical controls.

No outliers were detected; across the nine studies, standardized residuals ranged from −1.53 to 1.68. The weighted mean effect size was significant (Hedges’ *g* = 0.576, *K* = 9, 95% CI [0.343–0.809], *z* = 4.849, *p* < 0.001), indicating better mentalizing for non-clinical controls than patients with somatization. Effect sizes ranged from 0.141 to 1.240, with all effect sizes in the expected direction. Of the nine effect sizes, five reached statistical significance (Figure 2). Homogeneity analyses revealed a tendency toward heterogeneity (*Q*(8) = 14.210, *p* = 0.076, *I*^2^ = 43.702). Sensitivity analyses, performed by omitting one study at a time from the random-effects model, showed mean effect sizes ranging between 0.512 and 0.649, indicating a robust effect.

##### Publication bias

**Figure 3:**
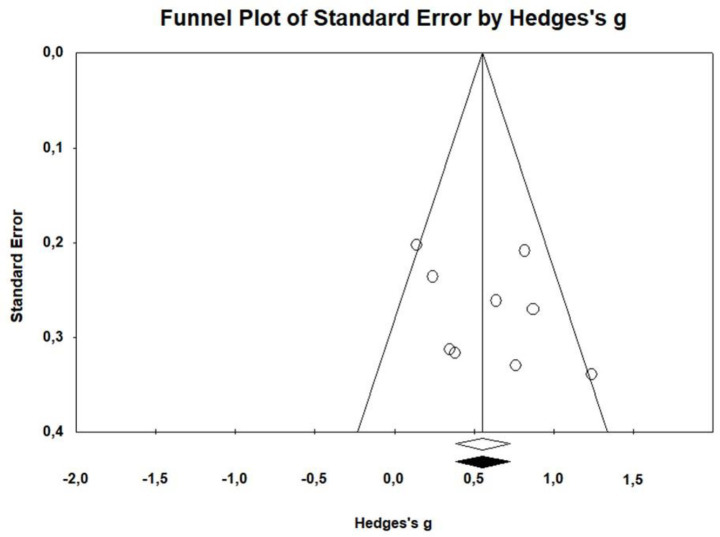
Funnel plot of the observed studies.

The Fail-Safe *N* analysis estimated that 91 additional studies would be required to nullify the overall effect size for differences between non-clinical controls and somatization groups. This exceeds the tolerance level (5 * *k* + 10 = 55), suggesting a robust effect. The funnel plot of observed and imputed studies (Figure 3) showed no missing studies according to Tweedie’s trim and fill results. Kendall’s tau (0.361, *p* = 0.175) and Egger’s regression intercept (2.719, *p* = 0.293) were not significant, indicating no evidence of publication bias.

##### Subgroup analyses

Because of the low number of contrasts (< 4), it was not possible to statistically compare subgroups. Therefore, we can only provide descriptive information for different subgroups.

##### Outcome

Across five studies assessing mentalizing about cognitive states (Schönenberg et al., 2014; Seitz et al., 2022; Subic-Wrana et al. (2010a); Thamby et al., 2019; Zunhammer et al., 2015), the weighted mean effect size was large and significant (Hedges’ *g* = 0.739, *K* = 5, 95% CI [0.457–1.020], *z* = 5.146, *p* < 0.001) and the effect was homogenous (*Q*(4) = 5.162, *p =* 0.271, *I*^2^ = 22.512). Across five studies assessing mentalizing about affective states (Cetin et al., 2021; Schönenberg et al., 2014; Seitz et al., 2022; Subic-Wrana et al. (2010a); Zunhammer et al., 2015) there was also a significant but medium effect (Hedges’ *g* = 0.594, *K* = 5, 95% CI [0.283–0.906], *z* = 3.743, *p* < 0.001), and the heterogeneity of the effect was not significance (*Q*(4) = 7.733, *p =* 0.102, *I*^2^ = 48.275).

##### Measure of mentalizing

In three studies (De Greck et al., 2012; Noll-Hussong et al., 2013; Ruckmann, 2015) using the self-report “Perspective taking” subscale (Davis, 1983), the weighted mean effect size was non-significant; the effect was homogenous (*Q*(2) = 0.554, *p =* 0.758, *I*^2^ = 0.000). In contrast, across six studies using mentalizing tasks (Cetin et al., 2021; Schönenberg et al., 2014; Seitz et al., 2022; Subic-Wrana et al. (2010a); Thamby et al., 2019; Zunhammer et al., 2015), the weighted mean effect size was significant (Hedges’ *g* = 0.725, *K* = 6, 95% CI [0.470–0.979], *z* = 5.587, *p* < 0.001), and heterogeneity was not significant (*Q*(5) = 7.093, *p =* 0.214, *I*^2^ = 29.505).

##### Types of mentalizing errors

Two studies explored the differences between SSD patients and non-clinical controls in overmentalizing and undermentalizing (Schönenberg et al., 2014; Seitz et al., 2022). For undermentalizing, the weighted mean effect size was significant (Hedges’ *g* = 0.518, *K* = 2, 95% CI [0.141–0.894], *z* = 2.695, *p* = 0.007); the effect was homogenous (*Q*(1) = 0.009, *p =* 0.924, *I*^2^ = 0.000). No significant weighted mean effect size was found for overmentalizing, and the heterogeneity of the effect was significant (*Q*(1) = 5.694, *p =* 0.017, *I*^2^ = 82.437).

##### Comparison of patients with SSD or corresponding diagnoses and clinical controls

Two studies (Cetin et al., 2021; Seitz et al., 2022) compared SSD patients to those with MDD or PTSD, yielding no significant differences.

##### Meta-regression analyses

Meta-regression analyses revealed no significant effects of publication year, study quality, mean age, female ratio. Altogether six studies reported means of depressive symptom scores for both patient and the control groups (Cetin et al., 2021; De Greck et al., 2012; Noll-Hussong et al., 2013; Seitz et al., 2022; Thamby et al., 2019; Zunhammer et al., 2015). Our meta-regression analysis showed no effect of depressive symptoms either.

Apart from depressive symptoms, unfortunately, no other comorbidities were assessed systematically in the selected studies. As shown in Table 2, two studies assessed anxiety, however using the different concepts of trait anxiety (Noll-Hussong et al., 2013) and generalized anxiety (Thamby et al., 2019). One further study assessed psychological distress and PTSD (Seitz et al., 2022). For this reason, it was not possible to include any other potential comorbidity into our meta-regression analyses.

## Discussion

The present systematic review and meta-analysis were conducted to explore the differences of mentalizing in SSD patients compared to non-clinical and clinical controls. Despite the relevance of mentalizing ability for difficult interpersonal relationships, including private life and healthcare settings, only few studies have examined the performance of this patient group in comparison to non-clinical, and even fewer to clinical controls. A significant degree of heterogeneity was observed across patient groups as well as measures of mentalizing.

The main finding of our systematic review and meta-analysis is the significant mentalizing impairment in patients with SSD and corresponding diagnoses compared to non-clinical participants in studies using mentalizing tasks, but not in those utilizing a self-report measure. This is in line with previous evidence showing self-report cognitive empathy scores only accounting for a negligible variance in behavioural cognitive empathy (Murphy and Lilienfeld, 2019), which calls into question the utility of self-report measures of mentalizing. There was no effect of mean age, female ratio or depressive symptoms on the significant difference of mentalizing between patients with SSD and non-clinical controls. It would be intriguing to compare the ability of the different mentalizing tasks to differentiate SSD patients from non-clinical controls. Unfortunately, this was not possible due to the small number of studies using the same task.

The present meta-analysis found no evidence for a significant difference between patients with SSD or corresponding diagnoses and any other psychiatric conditions. The main reasons for that were the methodological heterogeneity, the varying clinical control groups, and consequently, the small number of studies using a clinical control group with the same diagnosis as well as the lack of systematic assessment of psychiatric comorbidities in the SSD group. In line with the transdiagnostic view of mentalizing impairments, similarly to alexithymia (Pisani et al., 2021), a question for future research can be, if significant differences can be found if all the above methodological criteria are met. Unfortunately, the comparison of SSD to somatic conditions was not possible either, due to the lack of relevant studies.

Two studies using MASC reported the exact types of mentalizing impairment (Schönenberg et al., 2014; Seitz et al., 2022). Our meta-analysis showed a homogeneous effect of undermentalizing in patients with somatoform disorders or SSD compared to non-clinical controls, while no significant difference was found in overmentalizing. More studies using measures with the potential to assess different mentalizing errors are needed to test the robustness of this finding.

A further important finding is the effect of trauma history on the increased level of overmentalizing in somatic symptom disorders as well as major depression (Seitz et al., 2022). Trauma history was unfortunately not systematically assessed in the selected studies, with the exception of the most recent one (Seitz et al., 2022), although previous literature shows a link of trauma history to SSD (Achenbach et al., 2022; Doustmohammadi et al., 2024; Spitzer et al., 2008), as well as to mentalizing impairments (Nazarov et al., 2014; Simon et al., 2019). More specifically, trauma history has been associated with the necessity of activating more cognitive resources compared to non-clinical controls in order to perform well in situations relying on social cognition (Couette et al., 2022). Further studies exploring trauma history as well as using mentalizing tasks eligible for differentiating between different kinds of mentalizing errors are needed to gain more evidence of this potential connection.

### Implications for future research

The results of the included studies were heterogenous in terms of finding significant mentalizing impairments in certain diagnostic groups compared to controls, such as SFD; (Subic-Wrana et al., 2010a; Thamby et al., 2019) or PSPD; (Schönenberg et al., 2014; Zunhammer et al., 2015), but not in SSD (Seitz et al., 2022). More studies involving patients with the new diagnostic category of SSD would be necessary to tell if this was due to existing differences, small sample sizes, heterogeneity of measures, heterogeneity of patient groups or inclusion of both in- and outpatients in the meta-analyses. Future studies applying DSM-5 diagnostic criteria of the somatic symptom and related disorders would benefit from a more detailed description of specific diagnoses or symptoms of the included patients.

Our findings support the use of mentalizing tasks instead of self-report measures, especially those measuring specific aspects, such as mentalizing about cognitive and affective states as well as mentalizing errors. Findings regarding ROB show the need for more methodologically sound studies using mentalizing tasks with high validity and reliability, as well as larger samples of clinical and non-clinical controls. Additionally, further studies are needed to examine criterion validity of popular mentalizing tasks, in order to understand, how far the impairments measured by them relate to interpersonal difficulties in this patient group (Markiewicz et al., 2023). Further research would benefit from measuring executive functions parallel to social cognitive tasks in SSD as well as from a systematic assessment of comorbidities. Additionally, follow-up studies are needed to evaluate potential changes in mentalizing ability after completion of disorder-specific therapy.

### Strengths and limitations

We conducted a multinational systematic analysis, providing the first comprehensive overview of studies on mentalizing in patients with SSD and corresponding diagnoses in a controlled design. In our cohort, the diagnosis was validated by healthcare professionals. Another strength of this systematic review and meta-analysis is that mentalizing about both cognitive and affective states as well as the potential effect of depressive symptoms were analyzed.

However, our review process faced several challenges, especially the small number of relevant studies, heterogeneity in methods and the diverse clinical characteristics of the sample, such as the lack of systematic assessment of medication and comorbidities, which is a major limitation of our results. A further challenge was to summarize the results in different clinical populations with varying psychiatric diagnoses of earlier and recent studies, as previously diagnosed disorders do not align perfectly with new categories (Barsky, 2016) and their continuity is questionable (Ross, 2015). Furthermore, it is important to interpret our findings within the constraints of modest ROB management in the included studies, which is common in many cross-sectional designs (Wang and Cheng, 2020).

The mentalizing tasks used in the selected studies can be criticized based on methodological shortcomings including insensitivity to individual differences, ceiling effects and generally limited test reliability and validity (Yeung et al., 2024). The associations between observed measures have been found inconsistent, which raises questions about the comparability of results from different tasks (Yeung et al., 2024). Further, unlike real interpersonal encounters, some of the described mentalizing tasks lack dynamic, multimodal cues or contextual information (Achim et al., 2013; Msika et al., 2024). Participants are placed in an observer’s role, rather than actively engaging a social interaction (Msika et al., 2024). Additionally, the selected studies are lacking the exact description of comorbidities other than depressive symptoms. Consequently, it remains unclear, how far the mentalizing differences between patients and controls can be attributed to SSD or to the comorbidities.

## Conclusion

Our meta-analysis reveals that patients with SSD demonstrate significant mentalizing impairments compared to non-clinical controls, especially in mentalizing about cognitive states, with the tendency to undermentalize. Our findings can be interpreted in terms of the transdiagnostic view of mentalizing impairments. Reduced mentalizing in patients with SSD can lead to difficult interpersonal situations, amongst others, in healthcare settings, posing a therapeutic challenge in establishing a therapeutic alliance and contribute to the persistence of the disorder. Building up a validating relationship between healthcare professionals and patients (Johansen and Risor, 2017) and to avoid stigmatization and self-stigmatization (Brown, 2007) is crucial. A better understanding of the mentalizing abilities of this patient group may contribute to the reduction of their personal vulnerability and externalizing behaviors (Hijne et al., 2022).

## Data Availability

The data analyzed in this study is subject to the following licenses/restrictions: the dataset can be shared on request. Requests to access these datasets should be directed to krisztina.kocsis-bogar@meduniwien.ac.at.
