# Supplementary material for: Mentalizing impairments in somatic symptom disorder: a systematic review and meta-analysis
Source: Front Psychol. 2026 Feb 13;17:1704193. doi: 10.3389/fpsyg.2026.1704193 (PMC12947387; doi:10.3389/fpsyg.2026.1704193)
Supplement: Supplementary file 1 [file Supplementary_file_1.docx]

**Supplement 1**

**Search strategy using the example of MEDLINE**

1    exp "Theory of Mind"/
2    "theory of mind*".ti,ab,kf.
3    exp Empathy/
4    empath*.ti,ab,kf.
5    (emotion* adj3 aware*).ti,ab,kf,hw.
6    exp Mentalization/
7    mentalis*.ti,ab,kf.
8    mentaliz*.ti,ab,kf.
9    exp Social Cognition/
10    "social cognit*".ti,ab,kf.
11    1 or 2 or 3 or 4 or 5 or 6 or 7 or 8 or 9 or 10
12    Somatoform Disorders/
13    ("somatic symptom*" adj3 disorder*).ti,ab,kf.
14    somatoform*.ti,ab,kf.
15    exp medically unexplained symptoms/
16    (unexplained* adj3 symptom*).ti,ab,kf.
17    somatiz*.ti,ab,kf,hw.
18    somatis*.ti,ab,kf,hw.
19    "briquet syndrome*".ti,ab,kf.
20    Psychophysiologic Disorders/
21    ((psychosomat* or psychophysiolog* or psychoautonom* or somatopsych*) adj3 (disorder* or disturbance* or distort* or syndrome* or trauma* or factor* or pain* or symptom*)).ti,ab,kf.
22    exp Hypochondriasis/
23    hypochondr*.ti,ab,kf.
24    nosophob*.ti,ab,kf.
25    exp Body Dysmorphic Disorders/
26    (dysmorph* adj3 (disorder* or distort* or disturb* or dysfunct* or dysphoria* or body* or phob* or 'self image')).ti,ab,kf.
27    ("body image" adj3 (disorder* or distort* or disturb* or dysfunct* or dysphoria*)).ti,ab,kf.
28    dysmorphophob*.ti,ab,kf.
29    ((cardiac* or heart* or cordis*) adj2 (anxiety or neurosis* or phob*)).ti,ab,kf.
30    (cardioneurosis* or cardiophobia).ti,ab,kf.
31    "da costa syndrome".ti,ab,kf.
32    "effort syndrome".ti,ab,kf.
33    ((asthenia* or dystonia*) adj2 neurocirculat*).ti,ab,kf.
34    "neurogenic heart*".ti,ab,kf.
35    "functional heart complaint*".ti,ab,kf.
36    "gastr* neurosis*".ti,ab,kf.
37    (psychogen* adj3 (pain* or symptom*)).ti,ab,kf.
38    (pain* adj2 disorder*).ti,ab,kf.
39    (persist* adj2 pain*).ti,ab,kf.
40    psychalg*.ti,ab,kf.
41    exp Neurasthenia/
42    neurasthen*.ti,ab,kf.
43    psychasthen*.ti,ab,kf.
44    "illness anxiety disorder*".ti,ab,kf,hw.

45 “bodily distress disorder”

46 “bodily stress syndrome”
47    12 or 13 or 14 or 15 or 16 or 17 or 18 or 19 or 20 or 21 or 22 or 23 or 24 or 25 or 26 or 27 or 28 or 29 or 30 or 31 or 32 or 33 or 34 or 35 or 36 or 37 or 38 or 39 or 40 or 41 or 42 or 43 or 44 or 45 or 46
46    11 and 47
